# Redefining the Speed Limit of Phase Change Memory Revealed by Time-resolved Steep Threshold-Switching Dynamics of AgInSbTe Devices

**DOI:** 10.1038/srep37868

**Published:** 2016-11-25

**Authors:** Krishna Dayal Shukla, Nishant Saxena, Suresh Durai, Anbarasu Manivannan

**Affiliations:** 1 Discipline of Electrical Engineering, Indian Institute of Technology Indore, Indore - 453552, India; 2 Materials Science and Engineering, Indian Institute of Technology Indore, Indore - 453552, India

## Abstract

Although phase-change memory (PCM) offers promising features for a *‘universal memory’* owing to high-speed and non-volatility, achieving fast electrical switching remains a key challenge. In this work, a correlation between the rate of applied voltage and the dynamics of threshold-switching is investigated at picosecond-timescale. A distinct characteristic feature of enabling a rapid threshold-switching at a critical voltage known as the threshold voltage as validated by an instantaneous response of steep current rise from an amorphous off to on state is achieved within 250 picoseconds and this is followed by a slower current rise leading to crystallization. Also, we demonstrate that the extraordinary nature of threshold-switching dynamics in AgInSbTe cells is independent to the rate of applied voltage unlike other chalcogenide-based phase change materials exhibiting the voltage dependent transient switching characteristics. Furthermore, numerical solutions of time-dependent conduction process validate the experimental results, which reveal the electronic nature of threshold-switching. These findings of steep threshold-switching of ‘*sub-50 ps delay time*’, opens up a new way for achieving high-speed non-volatile memory for mainstream computing.

Chalcogenide-based phase-change materials have been successfully employed in various optical data storage products^1^,^2^ and have also recently demonstrated their capabilities in the next generation of high-speed non-volatile electronic memory^3^,^4^,^5^. Moreover, featuring a crystallization/re-amorphization speed of 500 ps by means of electrical priming^6^ and a high degree of scalability with an extremely low-power programming^7^ promises the so called *‘universal memory’* which could possibly replace almost all data storage devices^8^, including random access memories (RAM). Owing to this, a resurgence of interest has therefore been devoted towards realizing even ultrafast phase-change logic devices^9^,^10^.

In phase-change memory (PCM), information is encoded rapidly by means of switching between high-resistance amorphous (binary ‘0’) and low-resistance crystalline (binary ‘1’) phases owing to Joule heating caused by nano/pico-second (ns/ps) electrical pulses. Re-amorphized phase (reset) is achieved by voltage pulses having large amplitudes, which raise the local temperature above melting point within a duration as small as 400 ps^11^,^12^ owing to the conducting nature of the crystalline state. A rapid cooling subsequently takes-place during the sharp trailing edge, wherein the atoms are locked into a disordered state. However, to achieve a crystalline phase (set), a longer pulse-width of a few ns^5^,^11^,^12^ is required to essentially surpass an event of threshold-switching from amorphous off-to-on state^13^,^14^. Thereby, Joule heating causes the local temperature to rise above crystallization. Hence, the speed of crystallization achieved by set pulse is inherently governed by a combined effect of ultrafast threshold-switching dynamics and crystallization kinetics of phase-change (PC) material. These two key factors must therefore be discerned together when ultrafast crystallization is addressed.

Despite efforts to increase the crystallization speed by varying the amplitudes of applied voltage pulses systematically on several chalcogenide based PC materials^14^ such as GeTe^5^, Ge_2_Sb_2_Te_5_^11^,^12^,^15^,^16^, it has been difficult to realize speeds below 1 ns. This is primarily owing to the voltage dependent transient characteristics associated with threshold-switching process^11^,^14^. Hence the speed of threshold-switching is primarily dictated by transient parameters including delay time, t_d_, i.e. the time between voltage exceeding the threshold value V_T_ (onset) and the breakdown of electronic resistivity as exemplified by initiation of a steep rise in the device current (end) and switching time, t_s_ (from amorphous off-to-on state). Such characteristics primarily involve measurement of voltage and current simultaneously in order to reliably evaluate the transient parameters. Since the delay time decreases rapidly for over voltages^14^, the reported delay time value on the chalcogenide-based memory devices so far has only been in the order of 1–10 ns^5^,^12^,^14^,^17^,^18^ and the switching time is limited by the response time of the experimental setup^14^. Very recently a voltage-dependent threshold-switching dynamics in sub-ns timescale is demonstrated revealing a short delay time as small as 300 ps in InSbTe material probed using an advanced custom-built programmable electrical tester^19^. Owing to these facts, achieving a faster set process is primarily hindered by the voltage dependent transient parameters and therefore the speed of crystallization is much slower compared to that of amorphization, which is the main drawback keeping us from realizing ps-programming characteristics of PCM devices. Therefore, exploring novel materials with faster switching dynamics by means of a systematic understanding of threshold-switching dynamics and the crystallization process of PCM device together in ps-timescale is essential.

For testing memory cells with ps electrical pulses, carefully designed high-frequency contact-boards are usually employed that allow realization of time-resolved electrical quantities. However, in case of PCM, owing to threshold-switching a rapid change from its high resistance to a low resistance (from ~1 MΩ to a few 100 Ω) state causes loading and unloading of parasitic capacitances in ps, which limits realization of the actual response of the device. We tackled this key issue, by using a custom-designed programmable electrical tester (PET) having a dedicated measurement line to capture ultrafast transitions^19^. Therefore, this setup allows overcoming the experimental challenges significantly for a reliable exploration of speed limits of PCM devices (see Methods).

In the present study, we have chosen Ag-and In-incorporated Sb_2_Te (known as AIST) belonging to the second family of PC materials^1^ owing to its strikingly fast crystal growth velocities^20^ that are suitable for high-speed memory devices. PCM cells were fabricated so as to consist of AIST (80 nm) as the PC material sandwiched between Ti electrodes using RF sputter-deposition. Mechanical masks were used for creating AIST cells in a cross-bar like configuration^19^. To study the ultrafast switching dynamics, a custom-designed electrical tester^19^ was employed. In this setup, an arbitrary waveform generator allows electrical pulses down to a plateau length (duration of maximum voltage between the rising and falling edge), rise and fall time (i.e. time taken to reach voltage from 10% to 90% and 90% to 10% respectively) of 1 ns having an amplitude of up to 5 V and the digital storage oscilloscope is capable of capturing electrical transients at 50 ps resolution (time duration between two successive data points at a sampling rate of 20 GSa/sec).

## Results and Discussion

**Figure 1:**
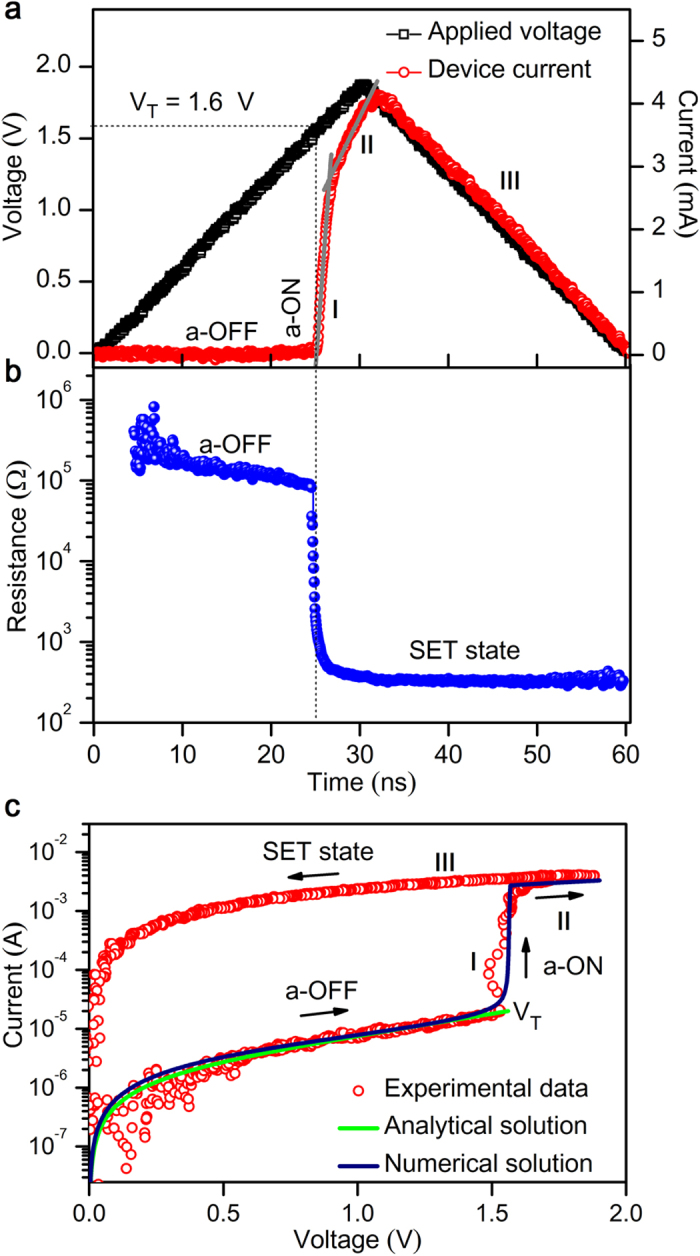
Time-resolved measurements of electrical switching dynamics. (**a**) Threshold-switching characteristics for V_A_ having an amplitude of 1.8 V and a leading/trailing edge of 30 ns. Threshold-switching occurs at V_T_ (1.6 V), the device current rapidly increases in two stages, (I- amorphous off-to-on state, and II-onset of crystallization) and during the trailing edge, I_D_ follows V_A_ revealing set transition (III-SET state). (**b**) Rapid change of dynamic resistances from high-resistance amorphous (~1 MΩ) to low-resistance (~300 Ω) crystalline state. (**c**) I-V characteristics of AIST cells show the features of amorphous off state in logarithmic current scale such that sub-threshold current increases linearly until a low voltage of 0.5 V above which the conductivity increases exponentially until threshold-switching at V_T_ (1.6 V). The conductivity rapidly increases above V_T_ in the on state, leading to set transition. The obtained experimental data was found to be in-agreement with analytical solutions^23^ in the sub-threshold conduction and also a good-match was found for the numerical solution based threshold-switching model^25^.

The electrical switching properties of several AIST cells are characterized in the as-deposited amorphous phase and all of these devices are initially at a high-resistance (R_OFF_ ~ 1 MΩ) off state. To understand the time-resolved current-voltage characteristics and switching behavior of AIST cells, a voltage pulse (V_A_) of amplitude 1.8 V with a leading/trailing edge of 30 ns is applied (Fig. 1a, black squares). During the leading edge of V_A_, the device remains in a high- resistance off state until a critical voltage called the threshold voltage (V_T_) of 1.6 V. Above this V_T_, the device current (I_D_) rapidly increases and leads to a conducting on state. Remarkably, the current response curve exhibits two different slopes of I_D_ (Fig. 1a, red circles), first a steep current-rise (marked as “I” in Fig. 1a), followed by a slower current-rise (marked as “II” in Fig. 1a) in the conducting state. During the trailing edge of V_A_, the formation of a low resistance state is maintained due to the crystallization of the conductive phase, so called set transition (marked as “III” in Fig. 1a). This rapid transition during threshold-switching can directly be demonstrated by an abrupt decrease in resistance from a high-resistance (~1 MΩ) amorphous phase to a low-resistance (~300 Ω) crystalline phase that is more than three orders of permanent change in magnitude (Fig. 1b). The measured V_T_ (corresponding critical electric field *E*_*T*_, of 20 V/μm) was found to be in good-agreement with literature^21^. Furthermore, the sub-threshold conduction behavior of amorphous off state are visible in a logarithmic current scale (Fig. 1c), which reveals that the sub-threshold current increases linearly for V_A_ up to 0.5 V and for higher voltages the conductivity increases exponentially until threshold-switching occurs at V_T_ (1.6 V). Thereafter the conductivity increases rapidly; hence an increase in current through the PCM cell marks the set transition. The obtained experimental data on sub-threshold conduction was found to be in-agreement with analytical solutions (Fig. 1c, green line) based on thermally assisted trap-limited conduction^22^,^23^ (see Supplementary Information). In addition to that, numerical calculations (Fig. 1c, blue line) were performed to validate the experimental data based on the field-controlled trap-limited conduction^24^,^25^,^26^, which confirm the consistency of the experimental data of sub-threshold conduction and electrical switching characteristics. Moreover, the parameters E_T_, I_T_, and electron temperature (T_e_) at threshold event were numerically calculated based on literature^25^ for AIST devices and these electrical quantities at E_T_ are found to be comparable with the experimental data (see Supplementary Information, Table S4).

For a precise measurement of the transient parameters that are associated with the electrical switching, a careful optimization of the applied voltage pulse parameters is essential. For instance, a steep leading edge (rise time, t_r_) enables determining the delay time precisely^11^,^27^, the pulse width (t_w_) controls the crystallization of the PC material^5^ and the trailing edge (fall time, t_f_) shows the status of device resistance. Therefore, for the present investigation to validate the dependency of transient parameters on applied voltage, pulses of various amplitudes such as 1.8 V, 2.1 V and 2.6 V having the pulse parameters of leading edge of 1 ns and pulse width/trailing edge of 100 ns were used.

**Figure 2:**
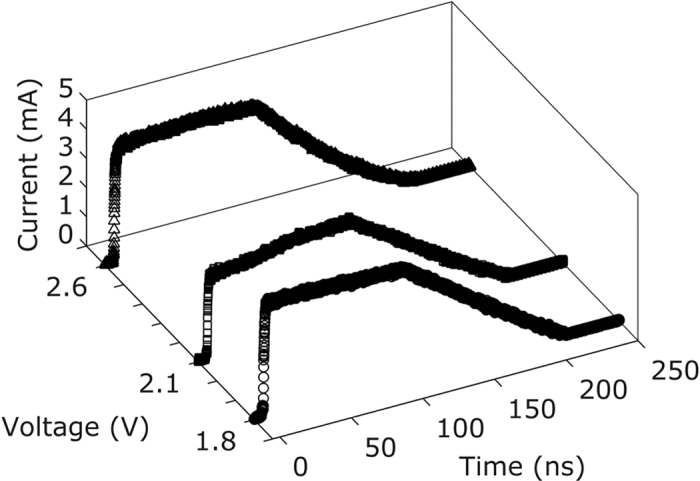
Time resolved measurement of device current. Variation of device current for three different V_A_ of amplitudes of 1.8 V, 2.1 V and 2.6 V having a rise time of 1 ns and a plateau/fall time of 100 ns.

The time-resolved ultrafast transient switching characteristics of numerous AIST cells examined here are in general delineated by the aforementioned specific pulse parameters. Figure 2 depicts the device current *I*_*D*_ measured for a V_A_ of 1.8 V, 2.1 V and 2.6 V. It was observed that AIST devices rapidly switch into a low-resistance conducting state and it persists during the trailing edge, which indicates the crystallization.

**Figure 3:**
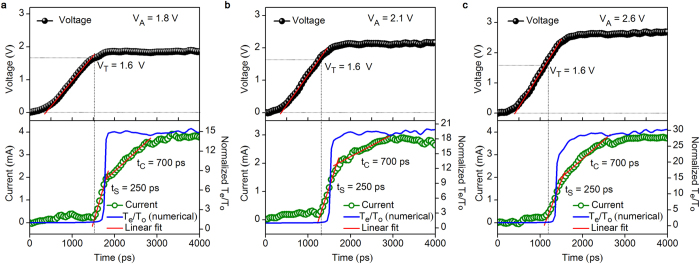
Threshold-switching dynamics. An enlarged view of V_A_ and I_D_ revealing transient threshold-switching dynamics in picosecond-timescale. Upon V_A_, I_D_ increases rapidly in two different phases. First, a steep current-rise occurs at a critical voltage called V_T_ (1.6 V) exhibited by threshold-switching from amorphous off-to-on state within the switching time (t_s_) of 250 ps, thereafter I_D_ increases with a distinctly lower slope until reaching saturation within 700 ps corresponds to crystallization time (t_c_) for different V_A_ (**a**) 1.8 V, (**b**) 2.1 V and (**c**) 2.6 V. The normalized carrier temperature (T_e_/T_o_) calculated using time-resolved numerical solution that clearly indicates that the T_e_/T_o_ remain unity until threshold-switching from the off-to-on state demonstrating an electronically governed threshold-switching mechanism. Thereafter T_e_/T_o_ increases rapidly depicting the onset of crystallization.

In order to shed light on the understanding of time-resolved transient switching characteristics in ps-timescale, we examined the dynamics of switching of AIST cells systematically using an enlarged view of V_A_ and I_D_ as depicted in Fig. 3 using time-resolved measurements. It can clearly be seen in Fig. 3a that upon encountering the leading edge of V_A_ (of 1.8 V), I_D_ increases rapidly in two different phases. First, a remarkable rapid threshold-switching occurs at a critical voltage called V_T_ (1.6 V) as exemplified by a steep current-rise indicating the breakdown of electronic resistivity without further measureable delay and thereafter switching from amorphous off-to-on state was achieved within the switching time (t_s_) of 250 ps. Subsequent to this, I_D_ increases with a distinctly lower slope until reaching saturation within 700 ps prescribed as the crystallization time (t_c_) of AIST PC materials^20^,^28^,^29^ revealing the set transition. Similar distinguishing characteristics of rapid threshold-switching and a steep current rise to conducting state within the stipulated t_s_ of 250 ps as well as current-rise at lower slope corresponding to t_c_ of 700 ps, were observed and found to be consistent with different V_A_ of 2.1 V (Fig. 3b) and 2.6 V (Fig. 3c). These results comprising the first experimental evidence of ultrafast threshold-switching at V_T_ in AIST devices is strikingly different from other PCM devices^5^,^11^,^13^,^14^,^30^. Moreover, switching from amorphous off to on state was achieved within a short time as small as 250 ps. This is approximately one order of magnitude faster than the threshold-switching speeds previously achieved in PCM devices^17^,^18^,^31^ and particularly GeTe-based PC materials^5^,^30^.

To further corroborate the nature of threshold-switching mechanism, the numerical solution for threshold-switching based on trap-limited conduction assisted by hot-electrons effects^25^ is computed using our experimental data. The normalized carrier temperature (T_e_/T_o_) is simulated using time-dependent experimental voltage (see Supplementary Information). We found that the signature of the T_e_/T_o_ is unity up to a steep current-rise from off-to-on state as initiated by threshold-switching even with various applied voltages (Fig. 3, blue lines). This confirms that the origin of threshold-switching is primarily governed by electronic mechanism^14^,^32^. Subsequent to these, when V_A_ is greater than V_T_, the rapid current-rise in the conductive state causes an abrupt increase in carrier temperature (T_e_ > T_o_), which indicates the initiation of the crystallization process. Although threshold-switching has long been recognized to be an electronic process with an intimate relation to the localized states, its detailed physical mechanism has been a subject for debate for more than four decades^14^,^18^,^22^,^25^. The present experimental findings and the numerical solutions reveal a direct evidence of steep threshold-switching governed by a purely electronic process. This electronic nature of threshold switching is supported by our simulation results where T_e_/T_o_ value is maintained unity until current reaches ~1 mA in the conducting state. Moreover, in addition to existing simulations results a direct experimental evidence of such feature may provide further clarity on understanding the nature of threshold switching mechanism which is a scope for future experiments. The observed speed of threshold-switching from amorphous off-to-on state is found to be 250 ps, which is at the verge of transient response time of the experimental setup (i.e. the rise time of contact-boards found to be 250 ps, see Methods as well as Supplementary Information, Fig. S2) indicating that even faster switching speeds could be achieved by improving the capabilities of the setup towards theoretical predictions.

**Figure 4:**
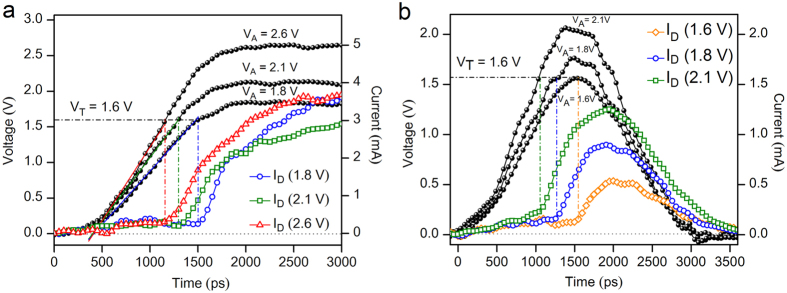
Ultimate speed of threshold-switching of AIST cells. (**a**) The signature of an instantaneous switching of steep current-rise at V_T_ of AIST cells was testified for various applied voltages of 1.8 V (1.13 V_T_), 2.1 V (1.3 V_T_) and 2.6 V (1.63 V_T_). A remarkable steep threshold switching is observed in AIST cells, indicating that the speed of threshold-switching is essentially achieved by the rate at which V_T_ is reached. (**b**) Rapid threshold-switching as exemplified by steep current-rise at V_T_ followed by crystallization process in AIST cells for V_A_ of 1.6 V, 1.8 V and 2.1 V having pulse width of 1.5 ns (FWHM).

Furthermore, the most crucial parameter that governs the speed of threshold-switching is t_d_, which exponentially decreases for increasing V_A_^13^. The smallest t_d_ achieved so far on GeTe, Ge_2_Sb_2_Te_5_ materials is only in the order of 1–4 ns^5^,^11^,^14^ and very recently a much smaller value of 300 ps^19^ in In_3_SbTe_2_ material is reported. Moreover, the delay time of GeSbTe and GeTe materials depend on the peak voltage of the pulse^5^,^6^, and therefore voltage-dependent switching characteristics of these materials require further research employing fast voltage pulses with higher amplitudes in order to measure lower delay times. Nevertheless, the voltage dependent t_d_ characteristics pose severe constraints on over voltages in order to minimize t_d_, which is a bottleneck to realize high-speed PCM for next generation computing. The present experimental data displayed in Fig. 4a reveals a strikingly different threshold-switching and a steep current-rise at critical voltage V_T_ (1.6 V) evidencing sub-50 ps delay time i.e. the data resolution of the DSO corresponding to sampling rate of 20 GSa/s (see Supplementary Information). The signature of an instantaneous switching of AIST cells at V_T_ was testified for various V_A_ of 1.8 V (1.13 V_T_), 2.1 V (1.3 V_T_) and 2.6 V (1.63 V_T_) (Fig. 4a, black solid circles). This confirms a remarkable steep threshold switching dynamics of AIST devices.

In order to further substantiate on steep threshold-switching characteristics in AIST cells, we used very short electrical pulses having pulse width of 1.5 ns (FWHM). Figure 4b demonstrates a similar threshold-switching characteristics of a steep current-rise at V_T_ as described above, for the V_A_ of 1.6 V, 1.8 V and 2.1 V. This further validates steep threshold-switching at V_T_ in AIST cells even for voltage pulses as short as 1.5 ns. It is also interesting to note that the ultimate speed of threshold-switching is essentially dictated by the velocity at which V_T_ is attained. Furthermore, it is noteworthy to mention here that crystallization was achieved within a short pulse width of 1.5 ns for V_A_ of 1.6 V, 1.8.V and 2.1 V as confirmed by a subsequent read pulse (see Supplementary Information).

Almost all of the chalcogenide thin film devices studied since 1960s^13^,^14^,^17^,^18^, including the first family of successful PC materials employed in optical storage, lie in the pseudo-binary line GeTe-Sb_2_Te_3_^1^ have shown significantly a voltage-dependent switching and transient characteristics^5^,^6^,^11^. On the other hand, this present study on AIST devices reveals a remarkable speed of threshold-switching which is primarily governed by the rate of V_T_ and it is independent to V_A_, i.e. threshold switching in AIST is independent of the rate at which the voltage is applied. This rate-independence is remarkable because it indicates that much shorter pulses could induce threshold switching at the same threshold electric field strength (E_T_). This unique switching property-portfolio of AIST devices is ideally suited for PC-RAM and also towards universal memory. There is further evidence in the form of the most successful optical memory products such as re-writable digital versatile discs (DVDs) and Blu-Ray discs that use AIST as the key material, which belongs to the second family of PC materials^1^ owing to its remarkable properties such as high-speed crystal growth velocities^21^,^31^,^33^,^34^ and easily reversible nature with better structural stability^1^,^29^,^35^. Furthermore, recent work^36^ on the relation between band gap and resistance drift in amorphous AIST material demonstrated a strikingly lower drift of the apparent activation energy compared with GeTe, GeSbTe materials. Therefore, such promising features of AIST material together with present experimental findings of unique threshold-switching dynamics with *‘sub-50 ps delay time’* substantiates in achieving a strikingly fast set state within an almost equal pulse width as that of reset state^6^,^11^,^12^ lead to a feasible solution to achieve a universal memory.

In conclusion, we have demonstrated the ability to control the ultimate speed of threshold-switching such that faster crystallization speeds can be accomplished. The rate-independent ultrafast threshold-switching dynamics of AIST devices investigated in this work is very different from other families of PC materials^1^,^2^. Hence, our findings of steep threshold-switching of AIST devices enable us to achieve not only the ultimate *‘universal memory’* for computing, but also pave the way to search novel materials suitable for gigahertz electronics.

## Methods

### Device Fabrication

The devices were fabricated on pre-cleaned (15 minutes of ultrasonic agitation each in acetone, isopropanol and dried with pressurized nitrogen) SiO_2_ substrates of size 20 mm × 20 mm by using mechanical masks as reported^19^. The cell structure consists of a phase-change material layer (80 nm of Ag_5_In_5_Sb_60_Te_30_, AIST), which is sandwiched between the top and bottom electrodes (55 nm of Ti). All these layers were radio frequency (RF) sputter deposited using sputter targets (99.99% purity) purchased from ACI Alloys, USA. First, a bottom electrode of Ti (55 nm) was deposited using 60 W RF power at 10 sccm Ar flow and 10 rpm substrate rotation with a sputtering rate of 0.0153 nm s^−1^. Subsequently, an active layer of AIST (80 nm) was deposited using 20 W RF power at 10 sccm Ar flow and 10 rpm substrate rotation with a deposition rate of 0.026 nm s^−1^. Finally, the top electrode of Ti (55 nm) was deposited with the same parameters as used for the bottom electrode. Mechanical masks were used to deposit various materials on the substrate in specific patterns^19^. The amorphous nature of as-deposited thin AIST films was confirmed using X-ray diffraction. The thicknesses of all films were measured using X-ray reflectometry and found to have variations within ±0.3 nm.

### Threshold-switching measurements and electrical characterization.

A custom-designed advanced programmable electrical tester (PET) with exceptional measurement capabilities at gigahertz (GHz) frequencies was employed for time-resolved electrical measurements at picosecond-timescale. PET comprises an arbitrary waveform generator (AWG, Agilent), a digital storage oscilloscope (DSO, Teledyne Lecroy), and a custom-made probe-station with GHz contact-boards having impedence matching circuits (IMC) and Amplifier circuits. The contact-boards consists of GHz compatible IMC using passive components that are compatible with frequencies up to 50 GHz having very small internal reactance (LC down to 1 × 10^−24^). The contact-boards provide two outputs simultaneously. One corresponds to the direct output line which reveals the ultrafast switching response of device from off-to-on state and the second output line diplays sub-threshold currents. All the measurements were made with a sampling rate of 20 GS/s having data resolution of 50 ps. In addition to this, it is important to note that capturing of ultrafast off-on transitions is primarily limited by the response time of the setup (i.e. rise time of contact boards found to be 250 ± 50 ps, see Supplementary Information, Fig. S2). Therefore, this setup is capable to identify switching transitions of devices from 250 ps onwards.

Temperature dependent thin film resistivity measurements on as-deposited thin AIST films were performed using the Van der Pauw technique. The samples were heated at the rate of 5 K min^−1^ in Ar atmosphere. The electrical resistivity decreases upon increasing the temperature and is found to exhibit a sharp reduction at 175 °C corresponding to the crystallization temperature (see Supplementary Information, Fig. S3).

### Theoretical analysis of threshold-switching using analytical and numerical solutions

An analytical solution of sub-threshold conduction was performed based on literature^23^. The subthreshold I-V curve shows a linear behaviour until a small applied voltage of 0.5 V, above which an exponential behaviour is observed. The obtained experimental data was found to be in-agreement with analytical solutions^23^. The numerical solution for threshold-switching^25^ was used to match the experimental data and parameters at the threshold event such as threshold voltage and threshold current were found to be in-agreement with analytical and numerical solutions (see Supplementary Information, Table S4).

## Additional Information

**How to cite this article**: Shukla, K. D. *et al.* Redefining the Speed Limit of Phase Change Memory Revealed by Time-resolved Steep Threshold-Switching Dynamics of AgInSbTe Devices. *Sci. Rep.* **6**, 37868; doi: 10.1038/srep37868 (2016).

**Publisher's note:** Springer Nature remains neutral with regard to jurisdictional claims in published maps and institutional affiliations.

## Supplementary Material

Supplementary Information
