# Supplementary material for: Redefining the Speed Limit of Phase Change Memory Revealed by Time-resolved Steep Threshold-Switching Dynamics of AgInSbTe Devices
Source: Sci Rep. 2016 Nov 25;6:37868. doi: 10.1038/srep37868 (PMC5122954; doi:10.1038/srep37868)
Supplement: Supplementary Information [file srep37868-s1.pdf]

Supplementary Information

Redefining the Speed Limit of Phase Change Memory Revealed by Time-resolved Steep Threshold-Switching Dynamics of AgInSbTe Devices

Krishna Dayal Shukla[1], **Nishant Saxena**[1], **Suresh Durai**[1] **and Anbarasu Manivannan**[1,2]*

[1]Discipline of Electrical Engineering, Indian Institute of Technology Indore, Indore - 453552, India

[2]Materials Science and Engineering, Indian Institute of Technology Indore, Indore - 453552, India

Correspondence and requests for materials should be addressed to A.M. (email:anbarasu@iiti.ac.in)

<u>SUPPLEMENTARY NOTE 1:</u> An advanced programmable electrical tester (PET) for nanoscale PCM device characterization:

Phase-change memory (PCM) devices undergo rapid and reversible structural change between amorphous to crystalline phases by means of sub-nanosecond (ns) electrical pulses[1-8]. Therefore, understanding their electrical switching dynamics and programming of nanoscale PCM device using picosecond (ps) electrical pulses require an advanced experimental setup with exceptional measurement capabilities at gigahertz (GHz) frequencies in order to address several challenges involved in PCM programming. These include a rapid change in resistances about more than three orders of magnitude during switching, which can cause loading/unloading of parasitic capacitance/inductance involved in the measurement line. Due to this, the response of switching dynamics of PCM device is restricted in the order of at least 1-50 ns[9]. Hence a high frequency compatible impedance matching circuit (IMC) is required in order to match the source and load resistances with the resistance of PCM along with overall electronics involved in the path[4,10].Therefore, an in-house advanced programmable electrical tester (PET) setup was designed to handle such numerous challenges during testing of PCM cells[11].

Calibration and performance analysis of PET:

At high frequency, several crucial factors such as noise, parasitic capacitance/inductance may affect electrical signals. This setup has been made with complete care to minimize these adverse effects. The performance of the setup is checked using the standard test pulse (STP) that is available with DSO having rise/fall time of 100 ps and pulse width of 100 ns with amplitude of 0.5 V. A systematic performance analysis was made on PET components such as high frequency cables and the contact-boards. All the high frequency cables were tested with STP and shown to have the rise/fall time of 100 ± 50 ps, which is almost same as that of STP within minimum (50 ps) variation as displayed in Fig. S1. Hence all the cables are capable to show step-response at least ≥ 100 ps. Furthermore, the contact-boards were tested with STP

and the rise time of contact board found to be 250 ± 50 ps as shown in Fig. S2 and hence, the contact-boards can reveal the step response of PCM cells at least ≥ 250 ps.

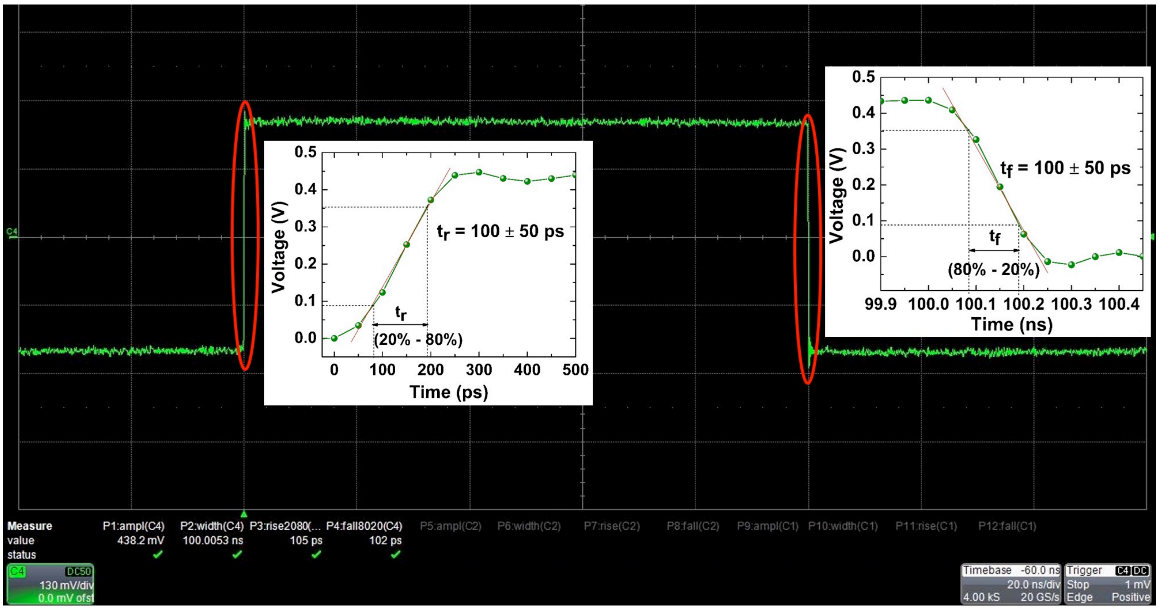

Figure S1. Step-response of high frequency cable for STP having rise/fall time of 100 ps and pulse width of 100 ns. The rise/fall time of cable is found to be 100 ± 50 ps.

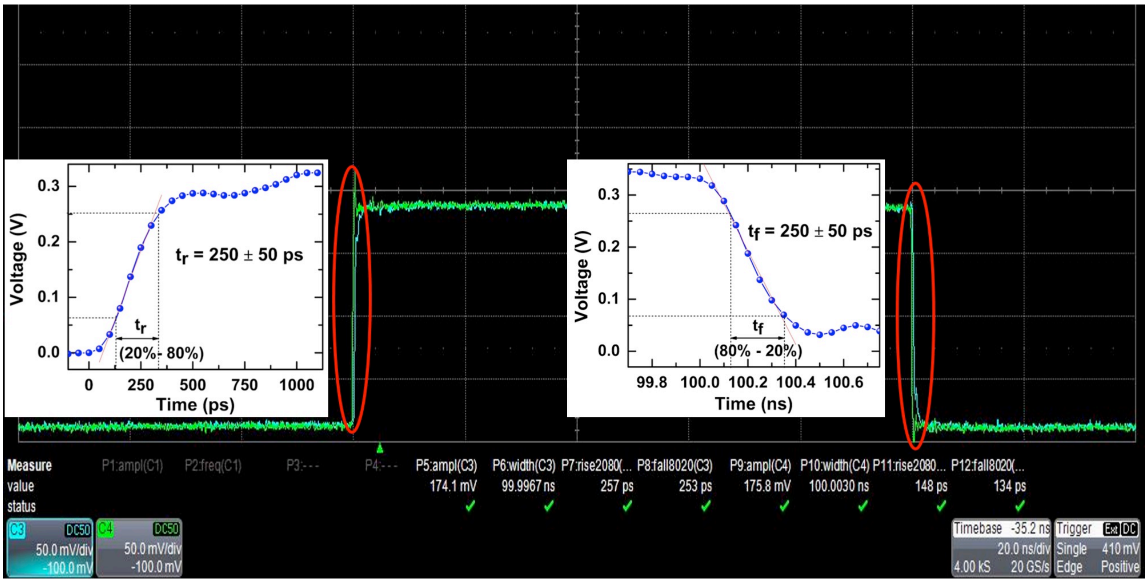

Figure S2. Step-response of high frequency PCB for STP having rise/fall time of 100 ps and pulse width of 100 ns. The rise/fall time of PCB connected using cables is found to be 250 ± 50 ps.

Temperature dependent resistivity measurement of AIST cells:

AIST phase-change material is known to have fast crystal growth velocities[1,3]. The temperature-dependent sheet resistance measurement of AIST is shown in Fig. S3. The measurement was performed in Ar environment with four-point probe configuration using "Temperature dependent Van-der-Pauw resistivity measurement setup". The as-deposited thin films are heated at the heating rate of 5 K min^{-1} and the resistivity decreases smoothly with increasing temperature, reflecting the semiconducting nature of the sample. For further increased temperature, a drastic drop occurs in their resistivity (nearly four orders of magnitude), owing to the phase change from the amorphous to the onset of crystallization. Crystallization temperature is found to be 175 ºC and is well matched with literature[14,15].

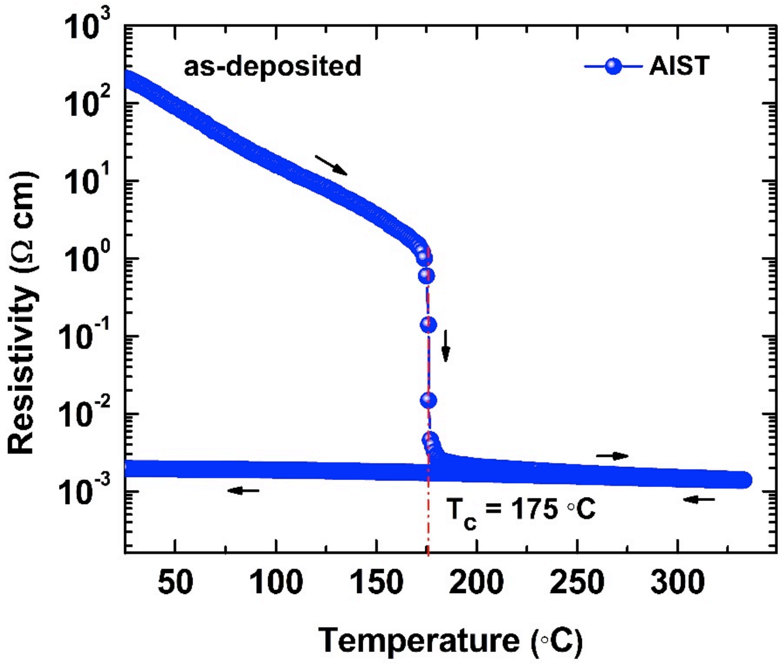

Figure S3. Temperature dependence of the resistivity for 140 nm AIST thin films (heating rate = 5 ºC/min). Dotted red line denotes the transition temperature T_c (175 ºC).

Time-resolved electrical switching measurements of AIST cells:

To identify a steady-state threshold voltage (V_T) of AIST cells, voltage pulse having amplitude of 1.8 V with rise/fall time of 30 ns is applied on several cells. The channel 1 (Ch1) of DSO measures the applied voltage, channel 2 (Ch2) and channel 3 (Ch3) measures the device switching response off-to-on state and the sub-threshold currents respectively. This test reveals V_T of 1.6 V ± 0.05 V. The obtained V_T of 1.6 V (equivalent threshold electric field (E_T, 20 V/μm is calculated across the device thickness) is in good agreement with reported elsewhere[16,17]. I-V characteristics of AIST cells show the features of amorphous off state in logarithmic current scale such that a Ohmic conductivity till a low voltage of 0.5 V above which the conductivity increases exponentially until threshold-switching at V_T (1.6 V). The conductivity rapidly increases above V_T in the on state, leading to set transition. The obtained experimental data was found to be in-agreement with analytical solution in the sub-threshold conduction and also a well-match was found for the numerical solution based threshold switching model. The theoretical analysis of these experimental data using analytical and numerical solutions is discussed in the next subsection.

Further, to study the ultrafast switching behavior of AIST cells, AWG is used to generate fast pulses with minimum rise/fall time of 1 ns, pulse width (full width half maximum, FWHM) of 1.5 ns and having amplitudes up to 5 V. DSO is used to capture the response of PCM device with the sampling rate of 20 GSa/s. In order to find the dependency of transient parameters such as delay time and switching time, voltage pulse with different amplitudes 1.8 V, 2.1 V and 2.6 V are applied. Transient parameters are found to be similar irrespective of applied excess voltage (higher than V_T). These values are mentioned in Table S1. Furthermore, threshold switching behavior is observed at V_T of 1.6 V, where a steep current-rise is seen for all the applied voltages such as 1.8 V, 2.1 V and 2.6 V. This clearly demonstrates that the threshold-switching behavior at V_T is independent of applied voltage and hence a steep threshold-switching is demonstrated at V_T within 250 ps for all the applied voltages.

Figure S4 shows a switching response of AIST cells including instantaneous threshold switching at V_T followed by a rapid crystallization process for electrical pulse having rise/fall time of 1ns and pulse width of 1.5 ns. The low resistance crystalline state was confirmed by read pulse (0.3 V, 100 ns pulse width).

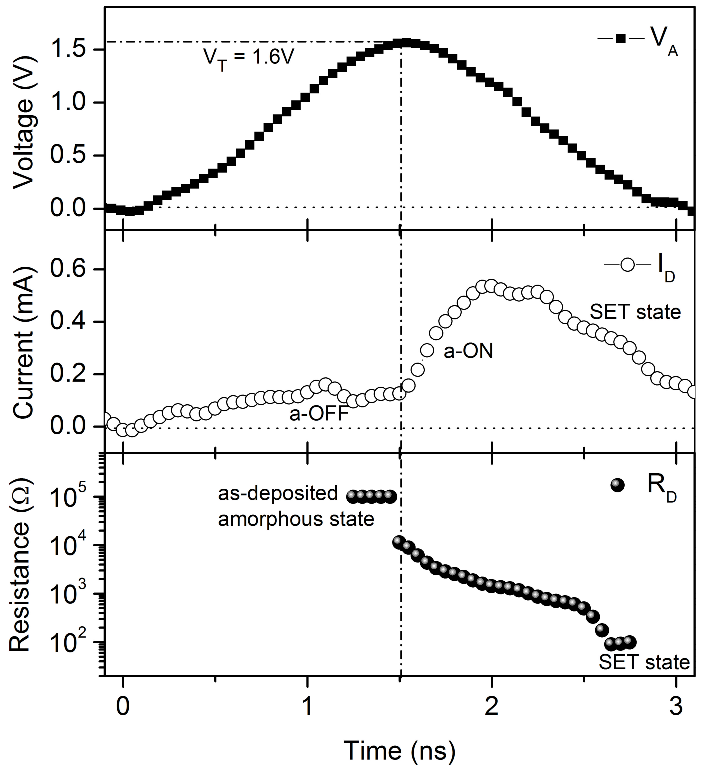

Figure S4. Ultrafast switching of *'sub-50 ps delay time'* and crystallization of AIST cells for V_A of 1.6 V and pulse width of 1.5 ns (FWHM) as represented by a steep current-rise and change in resistance permanently for more than 3 orders of magnitude representing *set* process.

Pulse amplitude	Switching time (t_s)	Crystallization time (t_c)
1.8 V	250 ± 50 ps	700 ± 100 ps
2.1 V	250 ± 50 ps	700 ± 100 ps
2.6 V	250 ± 50 ps	700 ± 100 ps

Table S1. Transient parameters of AIST device for pulses of different amplitudes

<u>SUPPLEMENTARY NOTE 2:</u> Theoretical analysis of experimental I-V curve using analytical and numerical solution:

Matching experimental I-V curve with subthreshold conduction model:

The subthreshold conduction model proposed for amorphous chalcogenides is based on trap limited conduction mechanism[18]. The conduction mechanism in the low current subthreshold regime ($V<V_{Th}$) is described as the thermally assisted hopping of carriers due to the significant concentration of localised states[18]. The subthreshold I-V curve shows a linear behaviour for smaller applied voltages and an exponential behaviour for larger voltages. The parameters used in the model are given in Table S2 and the equation used to calculate device current is given below:

$$I = 2qAN_T \frac{\Delta z}{\tau_o} e^{-(E_c - E_F)/kT} \sinh\left(\left(\frac{qV_A}{kT}\right)\frac{\Delta z}{2u_a}\right) \qquad (1)$$

where I is the current, q is the carrier charge, A is the contact area, N_T is the total trap density, Δz is the inter-trap distance, τ_0 is the carrier escape time, E_C-E_F is the activation energy, k is the Boltzmann constant, T is the temperature, V_A is the applied voltage and u_a is the thickness of amorphous region.

**Parameters**	**Values**
Activation energy (E_C-E_F)	0.315 eV
Inter-trap distance (Δz)	7 nm
Amorphous chalcogenide thickness (u_a)	80 nm
Total trap density (N_T)	2.5 x 10^{20} cm^{-3}
Escape time (τ_0)	1 x 10^{-15} s
Boltzmann constant (k)	8.629 x 10^{-5} eV/K
Temperature (T)	300 K
Carrier charge (q)	1.6 x 10^{-19} C

Table S2. Parameters used for analytical solution in sub-threshold regime as shown in Fig. 1c (green). Δz and N_T are the fitted parameters and K_B, T and q are the constants.

Matching experimental I-V curve with numerical solution based threshold switching model:

The numerical solution for threshold switching[19] is used to match an experimental I-V curve. In this model, bi-stability is described as trap limited conduction assisted by hot electron effects. At lower currents, most of the carriers occupy the trap states and giving a negligible contribution to the conduction. When increasing the current, the heated charge carriers occupy the band states for a significant conductivity. The simulated curve is obtained numerically by solving the below two supplementary equations as given in Ref. 19. Table S3 shows the parameters used in the numerical solution. The values of the parameters ΔE and n have been taken from the literature[20,21]. The parameters μ, τ_R and g_T/g_B have been tuned to match the experimental data.

$$JF = n\frac{k(T_e - T_o)}{\tau_R} \tag{2}$$

$$\frac{T_e}{T_o} = 1 + \frac{aF^2}{1 + (g_T / g_B)\exp(\Delta E / kT_e)} \tag{3}$$

where $a = \frac{\mu q \tau_R}{kT_o}$

where J is the current density, F is the electric field, n is the total carrier concentration, k is the Boltzmann constant, T_e is the electron temperature, μ is the mobility of the band carriers, τ_R is carrier relaxation time, g_T/g_B is the ratio of density of trap to band states, ΔE is the activation energy and T_0 is the temperature.

**Parameters**	**Values**
Activation energy (ΔE)	0.315 eV
Mobility of band carriers (μ)	6 $cm^2V^{-1}s^{-1}$
Carrier relaxation time (τ_R)	1.12 x 10^{-12} s
Ratio of density of trap to band states (g_T/g_B)	14 x 10^{-4}
Total carrier concentration (n)	1.45 x 10^{21} cm^{-3}
Amorphous chalcogenide thickness (u_a)	80 nm
Boltzmann constant (k)	8.629 x 10^{-5} eV/K
Temperature (T_o)	300 K
Electronic charge (q)	1.6 x 10^{-19} C

Table S3. Parameters used for numerical solution based threshold switching model for experimental data as shown in Fig. 1c (blue). μ, τ_R and g_T/g_B are the fitted parameters and K_B, T_o and q are the constants.

Parameters of interest at Threshold event:

The electron temperature (T_e^T), current (I_T) and electric field (E_T) at threshold event is calculated using analytical solution[19]. The equations 10 and 11 of an analytical solution mentioned in Ref. 19 are used to calculate the above parameters. Table S4 shows the calculated parameters at threshold event. T_e^T value obtained from analytical solution is in agreement with numerical solution.

**Parameters at threshold**	**Values**
Electron Temperature (T_e^T)	324.65 K
Current (I_T)	24.85 μA
Electric Field (E_T)	17.71 V.μm^{-1}
Voltage (V_T)	1.417 V

Table S4. Calculated numerical values of the parameters at threshold switching event using analytical solution[19].

References

1. Wuttig, M. & Yamada, N. Phase-change materials for rewriteable data storage. *Nat. Mater*. **6,** 824-832 (2007).
2. Lai, S. & Lowrey, T. OUM - A 180 nm Nonvolatile Memory Cell Element Technology For Stand Alone and Embedded Applications. *Tech. Dig. Int. Electron Devices Meet.* **36.5.1– 36.5.4** (2001).
3. Lankhorst, M. H. R., Ketelaars, B. W. S. M. M. & Wolters, R. A. M. Low-cost and nanoscale non-volatile memory concept for future silicon chips. *Nat. Mater*. **4**, 347-352 (2005).
4. Bruns, G. *et al*. Nanosecond switching in GeTe phase change memory cells. *Appl. Phys. Lett*. **95,** 043108 (2009).
5. Loke, D. *et al.* Breaking the Speed Limits of Phase-Change Memory. *Science* **336,** 1566-1569 (2012).
6. Cassinerio, M., Ciocchini, N. & Ielmini, D. Logic Computation in Phase Change Materials by Threshold and Memory Switching. *Adv. Mater.* **25,** 5975-5980 (2013).
7. Loke, D. *et al*. Ultrafast phase-change logic device driven by melting processes. *Proc. Natl. Acad. Sci. USA* **111,** 13272-13277 (2014).
8. Wang, W. J. *et al*. Fast phase transitions induced by picosecond electrical pulses on phase change memory cells. *Appl. Phys. Lett.* **93,** 043121 (2008).
9. Adler, D., Shur, M. S., Silver, M. & Ovshinsky, S. R. Threshold switching in chalcogenide glass thin films. *J. Appl. Phys.* **51,** 3289-3309 (1980).
10. Bruns, G., Electronic switching in phase-change materials. Ph.D. thesis, RWTH Aachen University (2012).
11. Pandey, S. K. & Anbarasu, M. Sub-nanosecond threshold switching dynamics and set process of In3SbTe2 phase change memory. *Appl. Phys. Lett*. **108,** 233501 (2016).

12. Adler, D., Henisch, H. K. & Mott, N. F. The mechanism of threshold switching in amorphous alloys. *Rev. Mod. Phys*. **50,** 209-220 (1978).

13. Buckley, W. D. & Holmberg, S. H. Electrical characteristics and Threshold Switching in amorphous semiconductors. *Solid-State Elect.* **18**, 127-147 (1975)

14. Njoroge, W. K. & Wuttig, M. Crystallization kinetics of sputter-deposited amorphous AgInSbTe films. *J. Appl. Phys.* **90**, 3816 (2001).

15. Hong, S. H., Bae, B. J. & Lee, H. Fast switching behavior of nanoscale Ag6In5Sb59Te30 based nanopillar type phase change memory. *Nanotechnology* **21**, 025703 (2010).

16. Krebs, D. *et al*. Threshold field of phase change memory materials measured using phase change bridge devices. *Appl. Phys. Lett*. **95,** 082101 (2009).

17. Koelmans, W. W. *et al.* Projected phase-change memory devices. *Nat. Commun.* **6,** 8181 (2015).

18. Ielmini, D. & Zhang, Y. Analytical model for subthreshold conduction and threshold switching in chalcogenide-based memory devices. *J. Appl. Phys.* **102,** 054517 (2007).

19. Buscemi, F. *et al*. Electrical bistability in amorphous semiconductors: A basic analytical theory. *Appl. Phys. Lett*. **104,** 022101 (2014).

20. Shportko, K. *et al.* Resonant bonding in crystalline phase-change materials. *Nat. Mater.* **7**,653–658 (2008).

21. Woda, M., Electrical transport in crystalline phase change materials, Ph.D. thesis, RWTH Aachen University (2010).
